# Incidence of bleeding during and after two non-surgical periodontal therapy schemes in patients with recent acute coronary syndrome on dual antiplatelet therapy: A pilot study

**DOI:** 10.4317/jced.61758

**Published:** 2024-12-01

**Authors:** Sandra Bibiana Moscoso, Fernán Mendoza, Luz Amparo Gómez, Andrea Londoño, Juan Sebastián Marín, Juan Manuel Sarmiento, Fabian Cortes, Paula Katherine Vargas-Sanchez, David Díaz-Báez, Gloria Inés Lafaurie

**Affiliations:** 1 Unit of Oral Basic Investigation, UIBO School of Dentistry, Universidad El Bosque, Bogota, Colombia; 2 Fundación Clinica Shaio, Bogotá 11001, Colombia; 3 Periodontics and Oral Medicine Program, School of Dentistry, Universidad El Bosque, Bogotá, Colombia

## Abstract

**Background:**

This study aimed to compare the incidence of bleeding using two periodontal treatment protocols in patients with recent Acute Coronary Syndrome (ACS)

**Material and Methods:**

This is an interim analysis of a double-blind controlled clinical trial evaluating two periodontal treatment schemes in patients with recent ACS treated with different dual antiplatelet regimens: Clopidogrel+ASA, Prasugrel+ASA and Ticagrelor+ASA. After randomisation six patients (22 quadrants) were treated with Scheme A (scaling and root planning-SRP) and six patients (21 quadrants) with Scheme B (ultrasonic scaling-US). Periodontal therapy was performed in two appointments using a preventive local protocol to avoid bleeding. The incidence of bleeding was evaluated at 30 minutes and 12 and 24 h later. The clot formation time and perioperative bleeding were also assessed and associated with the regimen using Chi-square/Fisher tests.

**Results:**

Profuse bleeding during treatment was significantly higher in patients with SRP 9/22 (40.91%) than in those with US 2/21 (9.52%) (*p* = 0.018). Intra-operatory bleeding in quadrants was major in SRP treated with Clopidogrel +ASA (*p* = 0.009). Only 2/12 patients presented with late bleeding after periodontal treatments, representing 16.6% per individual and 11.6% (5/43) per quadrant. However, the incidence of bleeding did not differ significantly between the two protocols.

**Conclusions:**

Post-treatment bleeding was moderate and similar in non-invasive and invasive periodontal treatment with different dual antiplatelet therapies. The periodontal treatment in patients with recent ACS treated with dual antiplatelet therapy is safe. The incidence of bleeding is low, and it can be controlled using local methods.

** Key words:**Bleeding, dual platelet anti-aggregation, periodontitis, acute coronary syndrome.

## Introduction

The American Heart Association (AHA) defines acute coronary syndromes (ACS) as a series of diseases that occur due to the rupture of an atheromatous plaque, which determines the production of a coronary thrombus that reduces blood flow to the heart (1). The treatment of patients with coronary events is based on outpatient cardiac rehabilitation and secondary prevention (CR/PS) programmes (2). Pharmacological management with anti-hypertensive medications is recommended in such patients; lipid-lowering drugs such as statins, dual antiplatelet therapy with aspirin and P2Y12 inhibitors, angiotensin-converting enzyme (ACE) inhibitors or angiotensin receptor blockers, mainly if they are hypertensive, diabetic or have poor ventricular function, beta-blockers after myocardial infarction and anticoagulants depending on specific cases (3). However, other antiplatelet therapies are currently used to treat patients with ACS (4).

As some practice guides indicate, the authors recommend postponing dental treatments until six months after a vascular event (5). Likewise, others propose that elective procedures that cause bleeding should be delayed until the risk associated with the discontinuation of antiplatelet or anticoagulant therapy is minimal (6,7). The perioperative management guidelines for patients on anti-coagulants and anti-platelets emphasise the evaluation of the individual risk of thromboembolic events and bleeding during and after any non-cardiac procedure (6).

Many patients with ACS do not receive periodontal treatment, possibly because practice guidelines have proposed postponing dental care (6,7). However, periodontal disease may be essential in determining recurrent cardiovascular events in patients with myocardial infarction (MI) (8). Likewise, patients with periodontitis who received periodontal treatment within a year following an MI event had a lower risk of recurrent cardiovascular events (9). In addition, Skaar *et al*. (2012) found no associations between dental invasive treatment at 30 and 180 days, including bacteraemia and a second vascular event (5).

The recommendations for patient care after an acute coronary syndrome (ACS) indicate that dental treatments (including periodontal treatment) are low risk in post-event patients if they are performed at least one month after the event when the patient has recovered his metabolic functions and is under medical recommendation (10). However, there is little evidence of postoperative bleeding with antiplatelet therapy in patients treated periodontally.

This study aimed to compare the incidence of bleeding in two periodontal treatment schemes in patients with ACS medicated with three different dual antiplatelet combinations.

## Material and Methods

-Study design and population

This interim analysis of a double-blind controlled clinical trial evaluated two periodontal treatment schemes in patients with stage II and III periodontitis with recent ACS (in the last two months) who were treated with different dual antiplatelet regimens. This study was approved by the Shaio Clinic Foundation Ethics Committee, Bogotá, Colombia (Act 276-19). The study was conducted per the ethical guidelines governing human health research from the Colombian Ministry of Health. Likewise, this study adheres to the Helsinki Declaration’s international ethical guidelines for human experimentation and all participants gave their written informed consent.

-Inclusion criteria

Patients admitted to the Shaio Clinic Foundation (Bogotá, Colombia) and treated per the guidelines for ACS. Patients aged ≤ 70 years who experienced an ACS event with or without ST elevation within the two months preceding the periodontal examination (11). Patients undergoing angioplasty with stent placement. Clinically stable patients (blood pressure <140/90-mm Hg, no angina, dyspnoea, or arrhythmias and oxygen saturation >90%). All patients were to be diagnosed with stage II and III periodontitis with at least two periodontal pockets in four quadrants for periodontal treatment, per the 2017 World Workshop on the Classification of Periodontal and Peri-Implant Diseases and Conditions (12).

-Exclusion criteria

Patients with uncontrolled diabetes, obesity, heart transplant, partially treated coronary disease, high risk of infective endocarditis, HIV, autoimmune diseases, history of acute infection in the last three months, surgically revascularized patients, patients who had been on antibiotic therapy in the last 3 months and anti-coagulants were excluded. Likewise, patients with less than six teeth in the mouth, those with poor oral conditions that could be exacerbated during follow-up and those who received periodontal treatment in the last six months were excluded.

-Study participants

**Figure 1:**
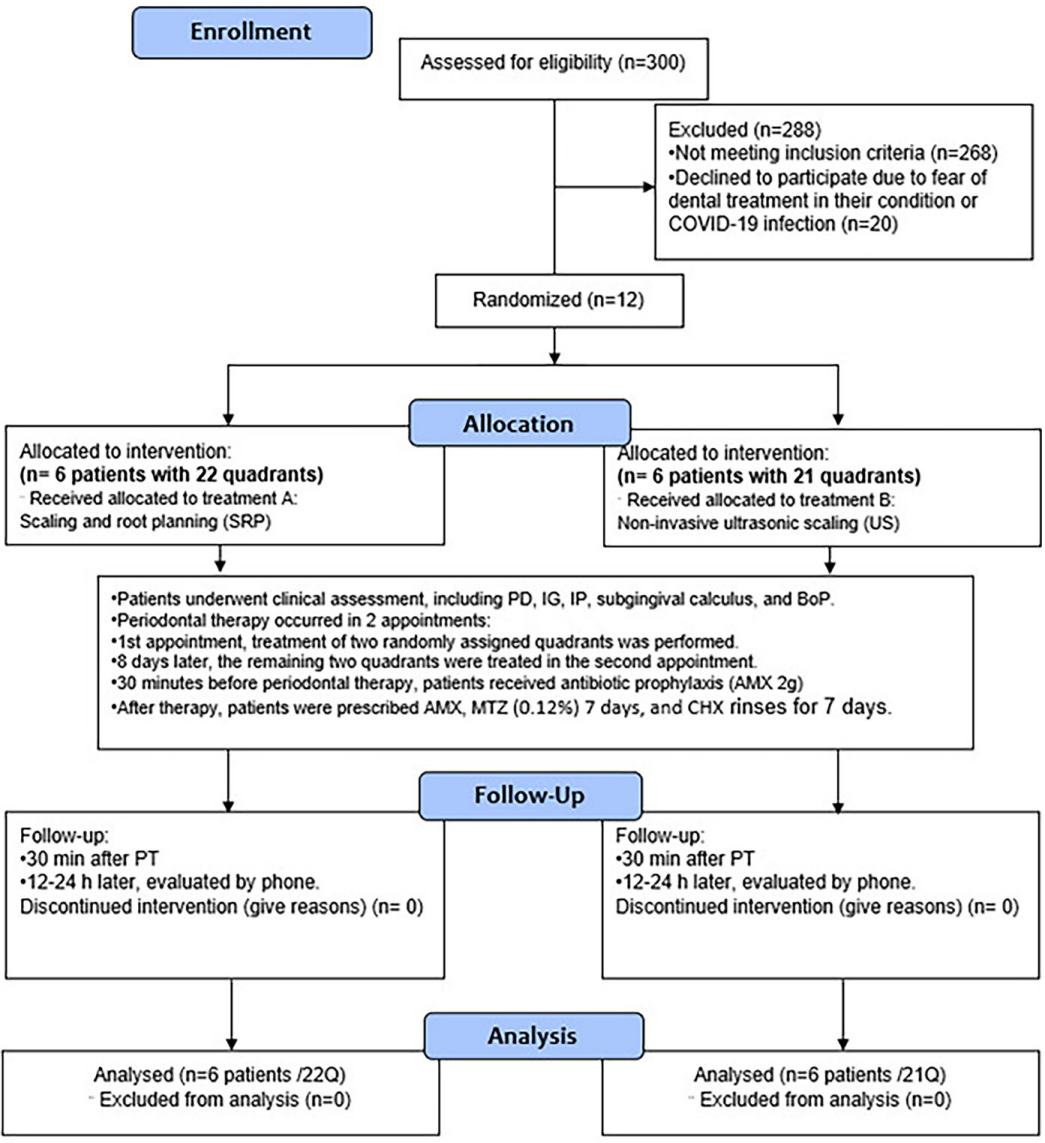
General design of the study (Consort flowchart adaptation for RCTs).

A blind assignment was performed in permuted blocks by an investigator external to the study group. Twelve patients were included in the randomised controlled trial pilot study, admitted to phase 2 of the cardiac rehabilitation programme of the Shaio Clinic (Bogota, Colombia) from June 2021 to June 2023, with a diagnosis of ACS and underwent stent placement. Two hundred and sixty-eight patients were excluded for different reasons; twenty eligible patients did not agree to participate due to fear of dental treatment or COVID-19 infection. (Fig. 1).

-Allocation concealment

The assignment of each participant to each treatment was hidden from the clinical researchers, coded with the letters A and B and delivered via email to the study headquarters, where the therapy was administered on the same day of each patient’s attendance. The research assistant obtained this information by preparing the required instruments per the protocol.

-Blinding

This was a single-blind study:

1) The patients were not blinded because they received two different treatments. However, the same numbers of appointments and treatments were assigned to each group, so they were observed similarly.

2) The clinical investigators were not blinded because they knew who received which treatment; however, the researcher who performed the clinical measurements was blinded to the clinical interventions.

3) The analysis was performed with coding and blinding was not undone until the analyses were completed.

-Medical protocol for ACS

The patients were administered a statin (atorvastatin 40 or 80 mg), angiotensin-converting enzyme inhibitors (ACEI), a beta-blocker, dual antiplatelet therapy with ASA and thienopyridines (Clopidogrel, Prasugrel and Ticagrelor). Patients also had a monitored exercise programme consisting of 36–40 sessions (2 h/week), risk factor control, nutritional and psychological therapy.

-Periodontal assessment

The clinical indices of each patient were assessed by a trained and calibrated periodontologist. The following periodontal clinical parameters were included: gingival index, plaque index, pocket probing depth (PD), clinical attachment level (CAL) and bleeding on probing (BoP). Patients had stage II and III periodontitis per the 2017 World Workshop on the Classification of Periodontal and Peri-Implant Diseases and Conditions (12).

-Periodontal treatments:

Twelve patients from the cardiovascular rehabilitation programme of the Shaio Clinic Foundation with ACS in the last two months who received different dual antiplatelet regimens (Clopidogrel +ASA, Prasugrel +ASA and ticagrelor +ASA) were evaluated. Six patients with 22 quadrants for treatment were assigned to treatment A, scaling and root planning (SRP) and six to Scheme B, non-invasive ultrasonic scaling (US) with 21 quadrants. All patients underwent anamnesis and assessment for the probing depth, gingival index, plaque index, presence of subgingival calculus and bleeding on probing. Periodontal therapy was performed in two appointments. In the first appointment, periodontal treatment of two quadrants was completed according to randomisation. Eight days later, the remaining two quadrants were treated at the second appointment. Thirty minutes before periodontal therapy, the patients were premedicated with antibiotic prophylaxis (2 g of amoxicillin). After treatment, they were given 500 mg of amoxicillin + 250 mg of metronidazole orally and chlorhexidine at 0.12% rinses for seven days.

-Bleeding incidence

The incidence of bleeding was evaluated according to the numbers of patients (n = 12) and quadrants (n = 43). This was studied as follows:

Early bleeding: 30 min after periodontal therapy.

-Late bleeding: Between 12 h and24 h later, evaluated by phone.

In addition to the incidence of bleeding, the following characteristics were evaluated during the perioperative period:

1) Amount of bleeding 

i. Minor bleeding: bleeding drop.

ii. Moderate bleeding: bleeding line.

iii. Severe bleeding: heavy bleeding.

2) Clot formation time in minutes.

3) Need for suture: If bleeding was profuse, suturing was required.

-Haemostasis protocols

All patients received a protocol of local haemostasis measures using continuous pressure with gauze for 15 min. After this time, if the initial haemostasis was not achieved, compression was performed for another 15 min. After completing this time and haemostasis, the patients were discharged with recommendations for a soft diet and gentle brushing for two days. They were given a package of sterile gauze with instructions on how to use it in case of early or late bleeding. The periodontist monitored each patient by telephone call to determine if any bleeding event occurred and, if required, a priority appointment to apply any of the three haemostasis protocols was realised with assignation sequential.

1) Applying pressure for 15 min with gauze impregnated with topical tranexamic acid prepared by macerating a 500-mg Tablet with physiological saline in the bleeding areas.

2) Applying pressure for 15 min with gauze impregnated with Aluminium Chloride on the bleeding areas.

suturing with 4-0 silk if bleeding has not stopped under the above measures.

-Statistical analysis

The incidence by quadrants and individuals was assessed. Frequencies of bleeding events were tabulated and compared between groups using the Chi-square and Fisher exact tests. The associations between bleeding and periodontal diagnoses and the anti-aggregation dual protocol were also evaluated using the Chi-square and Fisher exact tests with a significance level of 5%. The analysis was performed using Stata V14 (StataCorp LLC. USA).

## Results

 Table 1 shows the socio-demographic characteristics, anti-aggregation protocols and periodontal condition according to the periodontal treatment received. Sex and periodontal clinical parameters did not differ significantly between the groups (*p* < 0.05). However, despite randomisation performed, the group of patients treated with SRP was significantly younger than those treated with US (*p* = 0.01).

A total of 12 patients who were diagnosed with periodontitis between stages II and III were evaluated in a follow-up after two periodontal treatment schemes, without significant differences being observed in the periodontal diagnosis between groups. The patients had suffered a recent heart attack and were treated with dual antiplatelet therapy. Seven patients (58.4%) received Clopidogrel + ASA (25 quadrants), 4 (33.3%) received Ticagrelor + ASA (16 quadrants) and only one was prescribed Prasugrel + ASA (8.3%) (2 quadrants), and the differences among the groups were not statistically significant (*p* = 0.45).

-Incidence of bleeding in patients

There was no case of early bleeding in this study; no patient bled 30 min after the procedure. However, the amount of bleeding during the treatment tended to be greater in patients with RAR 4/6 than in those with ultrasound treatment 1/5 (*p* = 0.36). The type of periodontal treatment was not associated with the post-treatment bleeding time, immediate bleeding time or clot formation time > 15 min as evaluated by patients (*p* > 0.05).

Only 2/12 patients presented with late bleeding after periodontal treatment, representing a bleeding incidence of 16.6%. At 12 h, delayed bleeding occurred in 1/4 patients treated with non-invasive US and with the Ticagrelor + ASA combination (representing 8.3% of the cases with bleeding) and in 1/7 patients treated with SRP and with Clopidogrel + ASA (representing 8.3% of this complication). Prasugrel + ASA did not show any difficulties, although only one patient with two quadrants for treatment was admitted with this protocol until this analysis. At 24 hours, only one patient treated with US and Ticagrelor + ASA still showed postoperative bleeding despite having been treated with tranexamic acid as the haemostatic protocol. This patient’s bleeding was subsequently managed through sutures, after which he stopped bleeding.

-Incidence of bleeding by quadrants

The quadrants treated with the three antiplatelet regimens showed no significant differences in bleeding time (*p* = 0.89). However, the amount of moderate bleeding differed significantly for Prasugrel + ASA 2/2 (100%), followed by Ticagrelor + ASA 4/16 (25%) and less frequently for the quadrants treated with Clopidogrel + ASA 5/25 (20%) (*p* = 0.04). The analysis by treatment groups indicated that this bleeding occurred more frequently in patients treated with SRP; the amount of moderate bleeding during the procedure was significantly higher in patients with RAR 9/22 (40.91%) than in those with US 2/21 (9.52%) (*p* = 0.018). For comparisons between dual antiplatelet therapy protocols, this difference was significant only for clopidogrel in patients treated with SRP (*p* = 0.009) ( Table 2). Ultrasonic scaling required a suture after the procedure 3/21 (14.3%), which was inexistent in RAR (0/22). However, the type of periodontal treatment and anti-aggregation protocol was not associated with immediate post-treatment bleeding or with clot formation time after 15 min ( Table 2).

Late bleeding incidence at 12 h in the quadrants treated was 11.6% (5/43). After SRP, 2/22 (9%) and 3/21 (14.2%) patients in the US protocol had late haemorrhage treated with Clopidogrel and Ticagrelor, respectively. At 24 h, only three quadrants of the same patient treated with Ticagrelor + ASA and US showed postoperative bleeding despite having been treated with a haemostasis protocol (including compression and tranexamic acid) that required sutures.

## Discussion

In this pilot study nested to a randomised clinical trial, it was found that patients with recent acute coronary syndrome under dual antiplatelet therapy who had been diagnosed with periodontitis generated profuse bleeding during the procedure but did not have early bleeding after treatment. The incidence of later haemorrhage was mild during periodontal treatment, representing 16.6% by patient and 11.6% by quadrant. At 12 h, delayed bleeding occurred in one out of five patients treated with Ticagrelor + ASA, representing 8.3% of the cases with bleeding and in one out of seven patients treated with Clopidogrel + ASA, representing a similar percentage of this complication. Clopidogrel was one of the most used antiplatelet agents in this study and it caused slightly less intraoperative bleeding than Ticagrelor + ASA or Prasugrel + ASA. Generally, the most common anti-aggregatory treatments in patients treated with minor dental oral surgery are medicated with a single antiplatelet regimen such as aspirin, followed by clopidogrel and ticagrelor in a retrospective study; however, dual antiplatelet therapy represents the second frequency after ASA (13).

Several studies have also demonstrated a higher incidence of intraoperative bleeding in patients with prolonged dual antiplatelet therapy but with a low incidence of complications after dental extractions (14). An umbrella review of four systematic reviews of the impact of dual antiplatelet therapy on the increased risk of bleeding-related dental extractions showed that it could be reduced with adequate local haemostatic measures, and it is not necessary to interrupt systemic therapy during minor surgical treatment (15).

The incidence of bleeding after dental extractions in patients with dual antiplatelet protocols varies between 3.2% and 8.3%, which is slightly lower than that observed after periodontal treatment in this study (13). It is possible that periodontal treatment of an inflamed gum in recent ACS could have a more significant impact on postoperative bleeding, although the incidence is also low. However, per the findings of Rubino *et al*. in a retrospective analysis of 867 invasive periodontal procedures in patients treated with antiplatelet medications, oral anticoagulants or a combination of these drugs, the rate was low (0.35%). The number of patients treated with Prasugrel + ASA and ticagrelor + ASA was low in this study, and this could influence a lower incidence (16).

In this study, SRP was more susceptible to intraoperative bleeding than US. However, US required sutures after the treatment. The amount of post-treatment bleeding did not differ significantly between the two procedures. Therefore, it is essential to emphasise that non-invasive US has the same risk as SRP when periodontal treatments with the lowest risk of bleeding are selected for the treatment of patients with ACS.

Although clopidogrel was the most used medication in this study, the other protocols that used a new-generation P2Y12 receptor inhibitor (such as Ticagrelor + ASA or Prasugrel) are recommended in current therapeutic guidelines to treat patients with coronary heart disease. However, there is no evidence that their effectiveness exceeds that of Clopidogrel (17). Clopidogrel showed a lower intraoperative bleeding effect and better haemostasis in this study. In the Ticagrelor protocol, it was more challenging to control a late haemorrhage event in one patient. Nevertheless, since this study’s sample size is small, definitive conclusions cannot be drawn. However, Clopidogrel is recommended in patients aged >70 years because it has fewer bleeding complications in these individuals (18).

## Conclusions

This study demonstrates that it is possible to safely perform periodontal treatment in patients with recent ACS. Although some patients bled during treatment, the haemostatic protocol reduced the incidence of bleeding. Very few patients bled after periodontal therapy and these bleeds resolved without complications. The incidence of no-surgical periodontal therapy bleeding in antiaggregate individuals subjected to a strict local protocol for haemorrhage is mild and this does not justify the withdrawal of dual antiplatelet medication. Bleeding after procedures is late and can be overcome with local measures.

Patients with ACS are at risk of recurrence of coronary conditions associated with periodontal infection. Periodontal treatment may be required in patients with severe periodontal disease. non-surgical periodontal treatment could be performed even in patients medicated with dual antiplatelet therapy following a strict haemostatic protocol. The limitations of this study include its small sample size and the fact that it had to be suspended temporarily during the COVID-19 pandemic. However, many selected patients did not participate for fear of infection in the hospital or the risk of dental treatment due to their heart condition.

## Figures and Tables

**Table 1:** Sociodemographic characteristics and periodontal indexes at baseline of the study population according to periodontal treatment.

Variable	US	SRP	p-value
	n=6/21Q	n=6/22Q	
Gender F (%)			
Female	1 (16.6)	1 (16.6)	1
Male	5 (83.4)	5 (83.4)	
Medication patients			
Clopidogrel + ASA	4 (66.6)	3 (50)	0.45
Prasugrel + ASA	0 (0)	1 (16.6)	
Ticagrelor + ASA	2 (33.3)	2 (33.3)	
Medication (Q) F (%)			
Clopidogrel + ASA	13 (62)	12 (48)	0.36
Prasugrel + ASA	0 (0)	2 (9)	
Ticagrelor + ASA	8 (38)	8 (36)	
Diagnosis PDs F (%)			
Stage II periodontitis	3 (50)	3 (50)	1
Stage III periodontitis	3 (50)	3 (50)	
Age (years old)			
(Median IQR)	56 (52-62)	41 (37-59)	0.01*
No Teeth			
(Median IQR)	27 (26-28)	24 (17.5-28)	0.11
PI			
(Median IQR)	41.5 (15-75)	43 (3-88)	0.89
BoP			
(Median IQR)	19.5 (4-76)	34 (3-100)	0.42
PD			
(Median IQR)	2.45 (2.16-3.46)	2.35 (2.36-3.45)	0.49
CAL			
(Median IQR)	2.26 (1.92-3)	2.36 (1.6-3.2)	0.89

Q=Quadrant; F= Frequency; PDs= Periodontal Disease; IQR= Interquartile range (percentile 25-75); PI= Plaque Index (PI); BoP= Bleeding on probing; PD= pocket depht; CAL= Clinical attachment loss. US= Ultrasonic Scaling; SRP= Scaling and Root Planning.

**Table 2:** Incidence of immediate and late bleeding and clot formation by quadrants after periodontal treatment with different anti-aggregation protocols.

Variable	US		SRP		p-value
	n=21		n=22		
Intraoperative bleeding	Mild	Moderate	Mild	Moderate	
All anti-aggregant protocols	19(90.5)	2 (9.5)	13 (59.1)	9 (40.9) §	0.018
Clopidogrel + ASA	13 (100)	0 (0)	7 (58.3)	5 (41.6) §	0.009 NA NA
Prasugrel + ASA	0 (0)	0 (0)	0 (0)	2 (100)	
Ticagrelor + ASA	6 (75)	2 (25)	6 (75)	2 (25)	
Immediate Bleeding	Immediate	>15min	Immediate	>15min	
All anti-aggregant protocols	21 (100)	0 (0)	22 (100)	0 (0)	NA
Clopidogrel + ASA	13 (100)	0 (0)	12 (100)	0 (0)	NA NA NA
Prasugrel + ASA	0 (0)	0 (0)	2 (100)	0 (0)	
Ticagrelor + ASA	8 (100)	0 (0)	8 (100)	0 (0)	
Clot formation	<15min	>15min	<15min	>15min	
All anti-aggregant protocols	21 (100)	0 (0)	22 (100)	0 (0)	NA
Clopidogrel + ASA	13 (100)	0 (0)	12 (100)	0 (0)	NA NA NA
Prasugrel + ASA	0 (0)	0 (0)	2 (100)	0 (0)	
Ticagrelor + ASA	8 (100)	0 (0)	8 (100)	0 (0)	
Late Incidence	No	Yes	No	Yes	
All antiaggregant protocols	18 (85.7)	3(14.2)	20 (90.9)	2 (9.09)	0.59
Late Bleeding 12 hours	No	Yes	No	Yes	
Clopidogrel + ASA	13 (100)	0 (0)	11 (91.7)	1 (8.3)	0.28 NA 0.24
Prasugrel + ASA	0 (0)	0 (0)	2 (100)	0 (0)	
Ticagrelor + ASA	5 (62.5)	3 (37.5)	7 (87.5)	2(12.5)	
Late Bleeding 24 hours	No	Yes	No	Yes	
Clopidogrel + ASA	13 (100)	0 (0)	13 (100)	0 (0)	NA NA 0.055
Prasugrel + ASA	0 (0)	0 (0)	2 (100)	0 (0)	
Ticagrelor + ASA	5 (62.5)	3 (37.5)	8 (100)	0 (0)	

§ Significant differences between periodontal treatment *p*<0.05; ** Significant differences between anti-aggregant protocols. NA= It was not applied.

## Data Availability

The data that support the findings of this study are available from the corresponding author upon reasonable request.
